# Comprehensive quantitative analysis of vector beam states based on vector field reconstruction

**DOI:** 10.1038/s41598-019-46390-7

**Published:** 2019-07-10

**Authors:** Masato Suzuki, Keisaku Yamane, Kazuhiko Oka, Yasunori Toda, Ryuji Morita

**Affiliations:** 1 Department of Applied Physics, Hokkaido University, Kita-13, Nishi-8, Kita-ku, Sapporo, 060-8628 Japan; 2 Faculty of Science and Technology, Hirosaki University, 3 Bunkyo-cho, Hirosaki, 036-8561 Japan; 3 Laboratoire de photonique quantique et moléculaire, École Normale Supérieure Paris-Saclay, 61, avenue du Président Wilson, 94235 Cachan, Cedex France

**Keywords:** Applied optics, Optical physics, Other photonics, Optical physics

## Abstract

We demonstrate a comprehensive quantitative analysis of vector beam states (VBSs) by using a vector field reconstruction (VFR) technique integrating interferometry and imaging polarimetry, where the analysis is given by a cylindrically polarized Laguerre–Gaussian (LG) mode expansion of VBSs. From test examples of cylindrically polarized LG mode beams, we obtain the complex amplitude distributions of VBSs and perform their quantitative evaluations both in radial and azimuthal directions. The results show that we generated (*l*, *p*) = (1, 0) LG radially polarized state with a high purity of 98%. We also argue that the cylindrically polarized LG modal decomposition is meaningful for the detail discussion of experimental results, such as analyses of mode purities and mode contaminations. Thus the VFR technique is significant for analyses of polarization structured beams generated by lasers and converters.

## Introduction

Vector beam states (VBSs), which include vector vortex states or cylindrically polarized states^1^, are space variant polarization beam states having at least one polarization singularity and have been widely utilized in many applications such as laser processing^2–13^, laser manipulation^14,15^, quantum information processing^16–20^, classical communication^21–25^, microscopy^26–28^, and nonlinear spectroscopy^29–32^. In the present paper, we show that cylindrically polarized Laguerre–Gaussian (LG) mode expansions of VBSs are useful for comprehensive quantitative analysis and feasible by means of a vector field reconstruction (VFR) technique, which we introduce in the section of Reconstruction of Complex Amplitude Vector Distribution.

The most widely studied features of VBSs are the global polarization states (e.g., a radially polarized state) and the rotational symmetry of polarization distributions, that is, changes of local polarization states on the azimuthal axis. In addition, the complex amplitude vector distribution of VBSs on the radial axis has become known as an important property for several applications. For example, a radially polarized state with a nonzero radial index *p* can create a quite small spot of the longitudinal electric component on their propagation axis beyond the diffraction limit when it is tightly focused^33,34^. This feature enables refinement of material processing and super-resolution microscopy. Some researchers have reported that the complex amplitude distribution on the radial axis modulates the focal depth^35^ and the spot size^36,37^, the latter of which is vital for laser processing and manipulation technologies. We have reported that nonlinear propagations in a uniaxial crystal can alter the output complex amplitude distribution on the radial axis even if the input VBSs is unchanged^38^. That report implies that the nonlinear light-matter interactions may be controlled via the complex amplitude vector distributions of VBSs. Thus, technique for the full characterization of VBSs both in the radial and the azimuth axes is needed.

**Figure 1:**
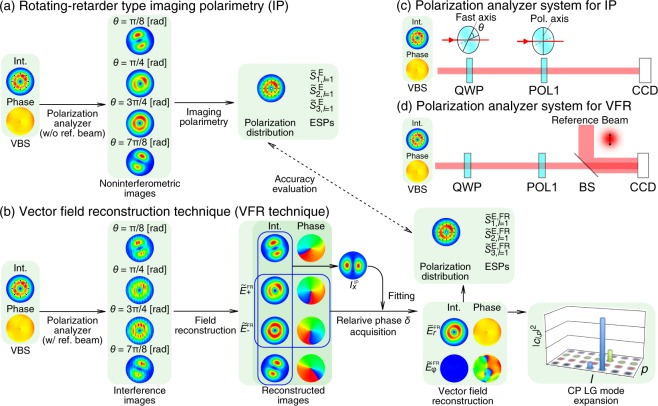
(**a,b**) Flow diagrams of (**a**) rotating-retarder type imaging polarimetry and (**b**) vector field reconstruction technique. (**c,d**) Experimental setup of polarization analyzer system for (**c**) imaging polarimetry and (**d**) vector field reconstruction. VBS, a vector beam state; QWP, a quarter-wave plate; POL, a polarizer; BS, a non-polarizing beam splitter; CCD, a charge coupled device camera (the pixel size was 6.45 *μ*m × 6.45 *μ*m). *θ* is the angle of the fast axis against the horizontal direction.

In the past, characterization of VBSs has been realized qualitatively by comparing the polarization-resolved intensity distributions with the calculated ones. Rotating-retarder type imaging polarimetry^39–41^ is a method to acquire polarization-resolved intensity distributions (Fig. 1(a)). By using a polarization analyzer system depicted in Fig. 1(c) ^40–42^, we capture four intensity images which are different in the angle of the quarter-wave plate in the system. The linear combinations of the four images represent the spatial distributions of Stokes parameters, that is, the polarization distribution of the object beam (See the section of Methods for the detail). For quantitative analysis, the authors established the extended Stokes parameters (ESPs) and their degree of polarization for the spatial distribution (DOP-SD), which enable us to directly evaluate the global polarization state and the rotational symmetry of a VBS, respectively^38,40,42,43^. However, these parameters, being calculated from just intensity distributions of several polarization components, do not provide a phase distribution. Moreover, information of VBSs with respect to the radial and azimuthal coordinates is lost because these parameters are spatially averaged. In order to make full fine evaluations of VBSs, we need to acquire complex amplitude vector distributions of VBSs.

The acquisition of complex amplitude vector distributions of VBSs has been reported by using a phase-shifting interferometry method^44–46^. This method requires taking multiple (over five) interference patterns. If an interferometer is not stable or arms are too long to keep the relative global phase fluctuate between the object and the reference beams, it is hard to correct the relative global phase of acquired interference patterns. The authors’ group has developed a field reconstruction technique for a uniformly polarized optical vortex (OV) state^47,48^. This field reconstruction technique gives a complex amplitude distribution of a certain uniform polarization component in the beam cross section. Since VBSs are expressed by the superposition of two orthogonal polarization components^49^, we can acquire the complex amplitude vector distribution of VBSs by taking the complex amplitude distributions in each orthogonal polarization through the field reconstruction technique (Fig. 1(b)). Figure 1(d) shows the system that integrates the imaging polarimetry and the field reconstruction, which is basically an extension of the polarization analyzer system introduced in Fig. 1(c). We call hereafter this technique reconstructing two orthogonal polarization states “the vector field reconstruction (VFR) technique”.

Moreover, VBSs can be decomposed in orthonormal basis such as cylindrically polarized Laguerre–Gaussian (LG) modes^41,50^. The cylindrically polarized LG modes, having two indices; the azimuth index *l* and the radial index *p*, are solutions to the paraxial Helmholtz wave equation. A single cylindrically polarized LG mode propagates with scaling its intensity and polarization distributions. As the LG modal expansion of a uniformly polarized OV state^48,51,52^, the mode expansion coefficients ${c}_{lp}^{r,\varphi }$ can be calculated from the complex amplitude vector distribution of VBSs. Here *r*, *ϕ* are radial and azimuthal coordinates, respectively. In the present paper, we expand VBSs into cylindrically polarized LG modes through an interferometric method.

We demonstrate cylindrically polarized LG modal expansion of VBSs by using a VFR technique based on interferometric field reconstruction, for the first time. The present paper is organized as follows. First, we describe a procedure of the VFR technique (Fig. 1(b)) in the section of Reconstruction of Complex Amplitude Vector Distribution. We experimentally demonstrate reconstructions of complex amplitude vector distributions of VBSs. The polarization distributions of the reconstruction results are compared with ones obtained by imaging polarimetry (IP) depicted in Fig. 1(a). Here, the IP method is regarded as VFR method without interferometry. Thus, we measured the object beams through both the IP method (blocking the reference beam) and the VFR technique by using the same optical setup. Second, we introduce a cylindrically polarized LG modal decomposition of VBS states in the section of Decomposition of Vector Beam State in Cylindrically Polarized Laguerre-Gaussian Modes, following which we show and discuss the decomposition results of the complex amplitude vector distributions. Finally, we summarize the conclusion in the section of Conclusion. We describe specific methods and definitions in the section of Methods.

## Reconstruction of Complex Amplitude Vector Distribution

### Principle of Vector field reconstruction technique

We describe the method of VFR technique. The VFR technique can be applied to paraxial beams with enough narrow bandwidth, whose electric field **E** is described by

1 $${\bf{E}}(r,\varphi ,z,t)=\tilde{{\bf{E}}}(r,\varphi ,z){\int }_{-\infty }^{\infty }\,f(\omega )\exp \{{\rm{i}}[k(\omega )z-\omega t]\}{\rm{d}}\omega ,$$

where *r*, *ϕ*, *z*, *t*, *ω*, and *k* give the radius from the beam center, the azimuthal angle in the beam cross section, the propagation axis, time, the angular-frequency, and the wavenumber, respectively, $\tilde{{\bf{E}}}(r,\varphi ,z)$ represents the complex amplitude vector distribution of a VBS, and *f*(*ω*) is a distribution function of angular frequency *ω*. Filtering the frequency domain by using a bandpass filter or a grating pair^47^, we can make VFRs of certain narrow frequency regions of broadband or ultrashort pulses as well as narrow bandwidth pulses.

The complex amplitude vector distribution $\tilde{{\bf{E}}}$ is resolved into two orthogonal polarization components, such as circular polarized components:

2 $$\tilde{{\bf{E}}}(r,\varphi ,z)={\tilde{E}}_{+}(r,\varphi ,z){{\bf{e}}}_{+}+{\tilde{E}}_{-}(r,\varphi ,z){{\bf{e}}}_{-},$$

where ${\tilde{E}}_{+}$ and ${\tilde{E}}_{-}$ represent the right circularly polarized (RCP) and the left circularly polarized (LCP) components, respectively. ${{\bf{e}}}_{+}={\mathrm{(1},{\rm{i}})}^{{\rm{T}}}/\sqrt{2}$ and ${{\bf{e}}}_{-}={\mathrm{(1},-{\rm{i}})}^{{\rm{T}}}/\sqrt{2}$ are the polarization basis for RCP and LCP states. As mentioned in introduction, the RCP and LCP components can be individually reconstructed by using the field reconstruction (FR) technique^47^, which was originally invented by Takeda *et al*.^53^. The FR technique is reviewed in the subsection of Field reconstruction of uniformly polarized optical vortex states, in Methods. Experimental setup for acquisition of interference pattern is shown in Fig. 1(c). We call the system a polarization analyzer system. The polarization analyzer system was based on a rotating-retarder type imaging polarimetor^39–41^, which was composed of a quarter-wave plate (QWP), a polarizer (POL1) and a charge coupled device (CCD) camera. A non-polarizing beam splitter (BS) was inserted between POL1 and the CCD camera in order to make interference between the VBS beam and a reference beam. When the reference beam is blocked, the polarization analyzer system is just a general imaging polarimeter. Thus, we can say that we integrated the imaging polarimeter with an interferometer into one system in order to reconstruct vector fields.

We can acquire the interference patterns for RCP and LCP components by setting the rotation angle $\theta =\pi \mathrm{/4}$ and 3*π*/4, respectively^42^, where *θ* is the angle of the fast axis of QWP from the horizontal direction (See the subsection of Field reconstruction of uniformly polarized optical vortex states, in Methods). When measurement is made at $z={z}_{0}$, the complex amplitude vector distribution on the measurement plane is described as follows:

3 $$\tilde{{\bf{E}}}(r,\varphi ,{z}_{0})={\tilde{E}}_{+}^{{\rm{FR}}}(r,\varphi ,{z}_{0}){{\bf{e}}}_{+}+{\tilde{E}}_{-}^{{\rm{FR}}}(r,\varphi ,{z}_{0}){e}^{{\rm{i}}\delta }{{\bf{e}}}_{-},$$

where ${\tilde{E}}_{+}^{{\rm{FR}}}$ and ${\tilde{E}}_{-}^{{\rm{FR}}}$ are the reconstructed RCP and LCP complex amplitude components, respectively. We note that the relative phase *δ* between RCP and LCP components cannot be generally neglected because it is not always true that the measurements of the interference patterns of the RCP and LCP components are made at the same time. Since we perform the measurements of the interference fringes in succession with a single CCD camera (Fig. 1(c)), which enables us to simplify the superposition of the RCP and LCP components without geometry transformation, we need to extract the relative phase *δ*. An earlier study^54^ acquired the interference patterns of *x*- and *y*-polarized components simultaneously by displacing them using a beam displacer. This manner can make reconstructing the complex amplitude vector distribution imprecise because there should remain the issue of spatial registration between *x*− and *y*− polarized components.

In order to extract the relative phase *δ*, it is sufficient to show the consistency in intensity distributions of a certain polarization component between IP and VFR methods. We estimated experimentally the relative phase *δ* through searching the minimum of the mean-squared error *G*_*δ*_ of intensity profile of the *x*-polarized component:

4 $${G}_{\delta }=\frac{1}{2\pi R}{\int }_{0}^{R}\,{\int }_{0}^{2\pi }\,{({|{E}_{x}^{{\rm{IP}}}|}^{2}-{|{\tilde{E}}_{+}^{{\rm{FR}}}+{\tilde{E}}_{-}^{{\rm{FR}}}{e}^{{\rm{i}}\delta }|}^{2})}^{2}{\rm{d}}r{\rm{d}}\varphi ,$$

where *R* is a cutoff radius, and ${|{E}_{x}^{{\rm{IP}}}(r,\varphi ,{z}_{0})|}^{2}$ is the intensity profile of the *x*-polarized component calculated through IP (Fig. 1(b)). The detail is described in the subsection of Evaluation of relative phase *δ*, in Methods. Finally, we acquire the complex amplitude vector distribution.

### Results and discussions

**Figure 2:**
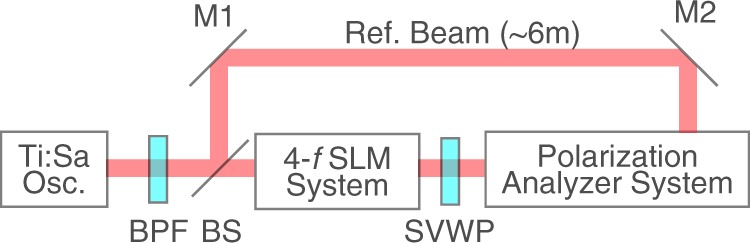
Experimental Setup. Ti:Sa Osc., a Ti:Sapphire oscillator; BPF, a bandpass filter; BS, a beam splitter; 4-*f* SLM system, a system of a spatial light modulator in the 4-*f* configuration; SVWP, a space vatiant wave plate; M1,2, mirrors; Polatization Analyzer System, a polarization analyzer system for the VFR method depicted in Fig. 1(d).

In this subsection, we report the analysis results of the cylindrically polarized LG pulses generated by using the combination of a 4-*f* spatial light modulator and a common-path optical systems in ref.^42^. Figure 2 shows an essence of the whole system. The detail of the generation system is shown in Fig. 8 of ref.^42^. The light source that we used was a Ti:Sapphire oscillator (central wavelength, 800 nm; bandwidth, 60 nm). A bandpass filter (central wavelength, 800 nm; bandwidth, 3 nm) narrowed the bandwidth so that we can apply VFR technique. A Mach–Zehnder interferometer was installed in the setup for VFR. One path was for the reference beam of the polarization analyzer system, the other path was for the generation of cylindrically polarized LG pulses. We generated the cylindrically polarized pulses with a complex amplitude modulation in the radial axis by using a spatial light modulator in the 4-*f* configuration^55,56^ and a space variant wave plate^57,58^.

In the present paper, we analyze two examples of the cylindrically polarized LG modes. The first one is $l=1$ radially polarized state with spatial modulation of a *p* = 0 LG mode (We call it *p* = 0 radially polarized LG pulses). The second one is $l=1$ radially polarized state with spatial modulation of a *p* = 1 LG mode (We call it *p* = 1 radially polarized LG pulses).

**Figure 3:**
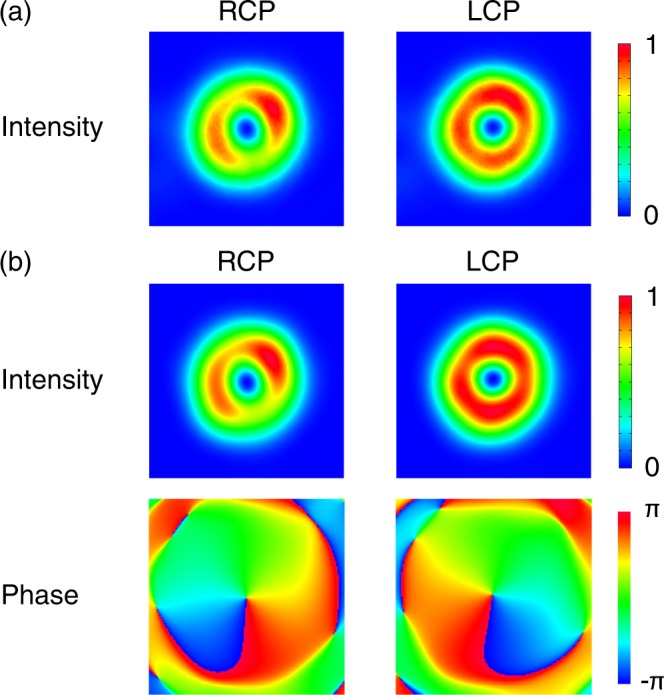
(**a**) Intensity distributions of RCP and LCP components captured by the IP method. (**b**) Intensity (upper row) and Phase (lower row) distributions of RCP and LCP components retrieved by the FR method. The sizes of images are 9.2 mm × 9.2 mm. Intensity values in (**a**) and (**b**) were normalized by the maximum intensity values of the LCP components in (**a**) and (**b**), respectively.

Figure 3 shows a comparison of the analysis results of *p* = 0 radially polarized LG pulses between the IP and FR methods. The intensity distributions of the RCP and LCP components measured by the IP method (i.e. a measurement without the reference beam in Fig. 1(c)) and the FR method are displayed in Fig. 3(a) and the upper row in Fig. 3(b), respectively. The intensity distributions were well reconstructed through the FR method. The fine noisy structure shown in the intensity distribution taken by the IP method (Fig. 3(a)) was not reproduced in the reconstructed ones thanks to the spatial frequency filtering in the reconstruction process. The mean-squared error of the RCP and the LCP intensity distributions were evaluated as 3 × 10^−4^ and 6 × 10^−4^, respectively. The low mean-squared errors (~10^−4^), which are the same orders as the previous study^47^, means that the reconstruction of the RCP and LCP components was well succeeded in. Unlike the IP method, the FR method outputs phase distributions (lower row in Fig. 3(b)) as well as the intensity distributions. From the phase profiles, the RCP and the LCP components were mainly *l* = −1 and $l=1$ OV states, respectively. That indicates that the VBS was mainly a $l=1$ cylindrically polarized state, that is, an axisymmetrically polarized (AxP) state^49^.

The arm length of the interferometer was over 6 m. Disturbance (e.g. vibration and air turbulence) easily led to a fluctuation of the arm length with respect to time, and therefore we needed to estimate the relative phase *δ* (see the subsection of Evaluation of relative phase *δ*, in Methods for the detail). From Eq. (4), we evaluated *δ* was 2.92 rad, thereby reconstructing the complex amplitude vector distribution.

Since the relative phase *δ* is a known parameter, we are able to change the basis from the cylindrically polarized ones to AxP ones:

5 $$\tilde{{\bf{E}}}(r,\varphi ,{z}_{0})={\tilde{E}}_{r}^{{\rm{FR}}}(r,\varphi ,{z}_{0}){{\bf{e}}}_{r}+{\tilde{E}}_{\varphi }^{{\rm{FR}}}(r,\varphi ,{z}_{0}){{\bf{e}}}_{\varphi }\mathrm{.}$$

**Figure 4:**
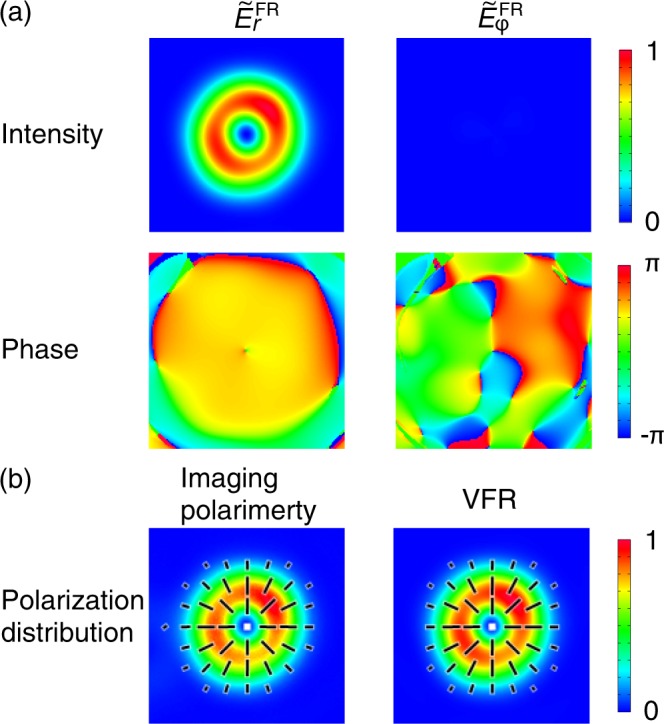
(**a**) Intensity (upper row) and Phase (lower row) distributions of ${\tilde{E}}_{r}^{{\rm{FR}}}$ and ${\tilde{E}}_{\varphi }^{{\rm{FR}}}$. (**b**) Comparison of polarization distributions derived from the IP method (left) and the VFR method (right). The black bars represent linear polarization and their directions are polarization axes of local linear polarization. The sizes of images are 9.2 mm × 9.2 mm. Intensity values were normalized by the maximum intensity value of ${\tilde{E}}_{r}^{{\rm{FR}}}$.

Here, **e**_*r*_ ≡ (cos*ϕ*, sin*ϕ*)^T^ and **e**_*ϕ*_ ≡ (−sin*ϕ*, cos*ϕ*)^T^ are, respectively, the basis for radially polarized and azimuthally polarized states. ${\tilde{E}}_{r}^{{\rm{FR}}}\equiv ({\tilde{E}}_{+}{e}^{{\rm{i}}\varphi }+{\tilde{E}}_{+}{e}^{{\rm{i}}(-\varphi +\delta )})/\sqrt{2}$ and ${\tilde{E}}_{\varphi }^{{\rm{FR}}}\equiv {\rm{i}}({\tilde{E}}_{+}{e}^{{\rm{i}}\varphi }-{\tilde{E}}_{+}{e}^{{\rm{i}}(-\varphi +\delta )})/\sqrt{2}$ are, respectively, the radially polarized and the azimuthally polarized components. Figure 4(a) shows intensity and phase distributions of the radially polarized and the azimuthally polarized components ($|{\tilde{E}}_{r}^{{\rm{FR}}}{|}^{2}$ and $|{\tilde{E}}_{\varphi }^{{\rm{FR}}}{|}^{2}$). Since the intensity of the azimuthally polarized component was small enough compared to the radially polarized one, the beam under test was regarded as a radially polarized state. We note that the phase distribution of the radially polarized component had no phase ramp along the azimuthal direction, which indicates the object beam under test was an AxP state. We discuss quantitatively it in the section of Decomposition of Vector Beam State in Cylindrically Polarized Laguerre–Gaussian Modes. We thereby accomplished the VFR of the object beam.

Since we know the complex amplitude vector distribution $\tilde{{\bf{E}}}(r,\varphi ,{z}_{0})$, we can obtain Stokes parameter on the measurement plane through the VFR method. Figure 4(b) gives polarization distributions of the beam obtained from the IP method (left) and the VFR method. The both polarization distributions represented the radially polarized state. It was hard to find the difference between them qualitatively. In order to make a quantitative comparison of the results between VFR and IP methods, we calculated the ESPs of the polarization distributions given by the IP method and the VFR method (${\tilde{{\bf{S}}}}_{l=1}^{{\rm{E}}}$ and ${\tilde{{\bf{S}}}}_{l\mathrm{=1}}^{E,\text{VFR}}$, respectively). Definitions of ESPs are described in the subsection of Extended Stokes parameters, in Methods and Refs. ^38,40,41,43^. Table 1 gives the values of ${\tilde{{\bf{S}}}}_{l=1}^{{\rm{E}}}$ and ${\tilde{{\bf{S}}}}_{l=1}^{E,\text{VFR}}$. The values of them were quite similar. The angle formed by the two extended Stokes vectors in the extended Poincaré sphere was $\arccos ({\tilde{{\bf{S}}}}_{l=1}^{{\rm{E}}}\cdot {\tilde{{\bf{S}}}}_{l=1}^{E,{\rm{V}}{\rm{F}}{\rm{R}}})=0.011$ [rad] = 0.60 [deg]. Thus, through the VFR method we reconstructed polarization distribution with an accuracy of 0.6 degrees on the extended Poincaré sphere.

**Table 1:** Values of ESPs of the *p* = 0 radially polarized LG polarization distributions obtained through the IP method (${\tilde{{\bf{S}}}}_{l=1}^{{\rm{E}}}$) and the VFR method (${\tilde{{\bf{S}}}}_{l=1}^{E,\text{VFR}}$).

	IP	VFR
${\tilde{S}}_{\mathrm{1,}l\mathrm{=1}}^{{\rm{E}}}$	0.999	0.999
${\tilde{S}}_{\mathrm{2,}l\mathrm{=1}}^{{\rm{E}}}$	−0.002	0.008
${\tilde{S}}_{\mathrm{3,}l\mathrm{=1}}^{{\rm{E}}}$	−0.046	−0.043

**Figure 5:**
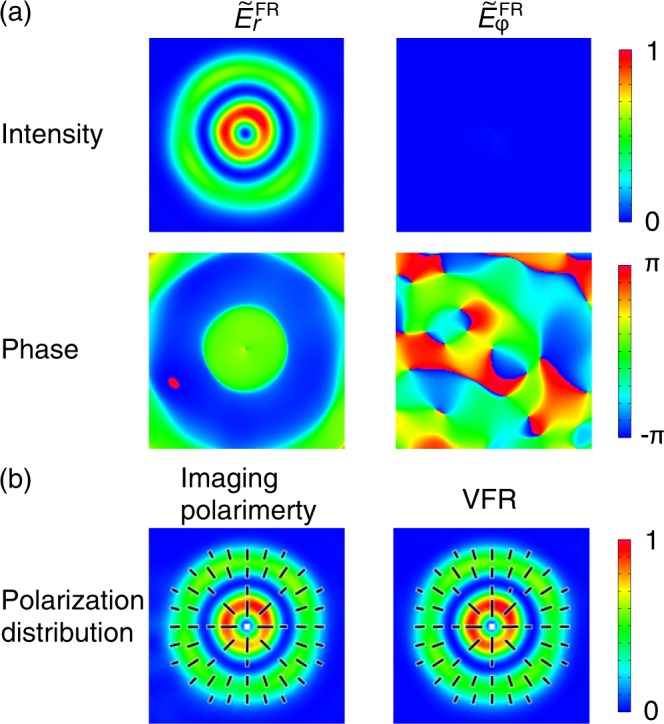
(**a**) Intensity (upper row) and Phase (lower row) distributions of ${\tilde{E}}_{r}^{{\rm{FR}}}$ and ${\tilde{E}}_{\varphi }^{{\rm{FR}}}$ of *p* = 1 radially polarized LG pulses. (**b**) Comparison of polarization distributions derived from the IP method (left) and the VFR method (right). The black bars represent linear polarization and their directions are polarization axes of local linear polarization. The sizes of images are 10.5 mm × 10.5 mm. Intensity values were normalized by the maximum intensity value of ${\tilde{E}}_{r}^{{\rm{FR}}}$.

In the same way, we investigated *p* = 1 radially polarized LG pulses generated by the system in ref.^42^. Figure 5(a) shows intensity and phase distribution for ${\tilde{E}}_{r}^{{\rm{FR}}}$ and ${\tilde{E}}_{\varphi }^{{\rm{FR}}}$. The intensity of ${\tilde{E}}_{\varphi }^{{\rm{FR}}}$ was almost null, thus the beam under the test was regarded as a radially polarized state. The intensity distribution of ${\tilde{E}}_{r}^{{\rm{FR}}}$ had two rings, which indicated a *p* = 1 LG mode. It is well known that there is a *π* phase shift between the inner and the outer rings of *p* = 1 LG mode^59^. The phase distribution of ${\tilde{E}}_{r}^{{\rm{FR}}}$ in Fig. 5(a) showed that the phase jump of ~π was located on the boundary of the inner and outer rings. The quantitative mode decomposition results is described in the section of Decomposition of Vector Beam State in Cylindrically Polarized Laguerre–Gaussian Modes. The polarization distribution derived from the IP method and the VFR method are displayed in Fig. 5(b). These polarization states were radially polarized ones. Table 2 gives the corresponding ESPs for the polarization distributions. The angle formed by the two ESPs was evaluated to be $\arccos ({\tilde{{\bf{S}}}}_{l=1}^{{\rm{E}}}\cdot {\tilde{{\bf{S}}}}_{l=1}^{E,{\rm{V}}{\rm{F}}{\rm{R}}})=0.0010$ rad = 0.059 deg, which shows that we excellently reconstructed the polarization distribution through the VFR method.

**Table 2:** Values of ESPs of the *p* = 1 radially polarized LG polarization distributions obtained through the IP method (${\tilde{{\bf{S}}}}_{l\mathrm{=1}}^{{\rm{E}}}$) and the VFR method (${\tilde{{\bf{S}}}}_{l\mathrm{=1}}^{E,\text{VFR}}$).

	IP	VFR
${\tilde{S}}_{\mathrm{1,}l\mathrm{=1}}^{{\rm{E}}}$	1.000	0.999
${\tilde{S}}_{\mathrm{2,}l\mathrm{=1}}^{{\rm{E}}}$	−0.003	0.003
${\tilde{S}}_{\mathrm{3,}l\mathrm{=1}}^{{\rm{E}}}$	−0.013	−0.012

Several research groups have reported the fiber mode expansion after a propagation in multimode fiber. Shapira *et al*. demonstrated the decomposition of eigenmodes of a photonic-band gap fiber through a noninterferometic approach^60^. This approach needs taking into account both of far-field and near-field intensity profiles. The issue of spatial registration^61^ (e.g. the uncertainty of beam centers) can easily decrease the accuracy of the decomposition results, and it is inevitable for this approach. Fatemi *et al*. reported LP mode decompositions of vector beams via an interferometric approach^54^. However, it is hard to restore the original complex amplitude vector distribution in the beam cross section because each polarization component is measured after spatial separation of two orthogonal polarization states and there also can remain the issue of spatial registration. In contrast to them, we can reconstruct complex amplitude vector distributions without the issue of spatial registration since our proposed method does not, in principle, need to capture images at different propagation positions or spatially separate the optical path of the two orthogonal polarization states.

## Decomposition of Vector Beam State in Cylindrically Polarized Laguerre–Gaussian Modes

### Description of mode decomposition

We here describe a cylindrically polarized LG mode decomposition. Since the cylindrically polarized LG modes ${u}_{l,p}^{{\rm{CPLG}}}$ are one of the orthonormal basis for paraxial beams, a complex amplitude vector distribution at the measurement plane can be decomposed as follows:

6 $$\tilde{{\bf{E}}}(r,\varphi ,{z}_{0})U(R-r)={E}_{0}\sum _{l,p}\,{u}_{l,p}^{{\rm{CPLG}}}(r,\varphi ,{z}_{0})({c}_{l,p}^{r}{{\bf{e}}}_{l}^{r}+{c}_{l,p}^{\varphi }{{\bf{e}}}_{l}^{\varphi }),$$

where

7 $${{\bf{e}}}_{l}^{r}=(\begin{array}{c}\cos \,l\varphi \\ \sin \,l\varphi \end{array}),\,{{\bf{e}}}_{l}^{\varphi }=(\begin{array}{c}-\sin \,l\varphi \\ \cos \,l\varphi \end{array}),$$

are the *l*th cylindrically polarized basis^43^ of radially polarized and azimuthally polarized states, respectively. *U*(*x*) is a step function that if *x* ≥ 0, *U*(*x*) = 1, otherwise *U*(*x*) = 0. Here, *R* is the cutoff radius introduced in Eq. (4). *U*(*R* − *r*) means that we put an imaginary aperture whose radius is *R* at the measurement plane. *E*_0_ represents an amplitude of the complex amplitude vector distribution, defined by

8 $${E}_{0}={[\frac{1}{\pi {R}^{2}}{\int }_{0}^{R}{\int }_{0}^{2\pi }(|{\tilde{E}}_{+}^{{\rm{FR}}}{|}^{2}+|{\tilde{E}}_{-}^{{\rm{FR}}}{|}^{2}){\rm{d}}S]}^{\mathrm{1/2}}\mathrm{.}$$

**Figure 6:**
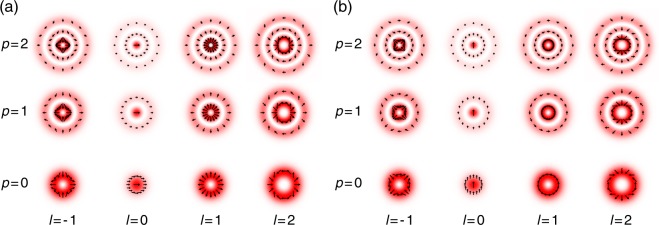
Electric-field vector and intensity distributions for *l* = −1 to *l* = 2, *p* = 0 to *p* = 2 (**a**) radially polarized modes ${u}_{l,p}^{{\rm{CPLG}}}{{\bf{e}}}_{l}^{r}$ and (**b**) azimuthally polarized modes ${u}_{l,p}^{{\rm{CPLG}}}{{\bf{e}}}_{l}^{\varphi }$. The vector directions give the relative relationships of the directions of electric vectors.

${c}_{l,p}^{r}$ and ${c}_{l,p}^{\varphi }$ in Eq. (6) are the mode coefficients for *l*th radially polarized and azimuthally polarized LG modes with a radial index *p*, respectively. Electric-field vector distributions and intensity distributions (${u}_{l,p}^{{\rm{CPLG}}}{{\bf{e}}}_{l}^{r}$ and ${u}_{l,p}^{{\rm{CPLG}}}{{\bf{e}}}_{l}^{\varphi }$) of radially and azimuthally polarized LG modes are displayed in Fig. 6(a,b), respectively. A definition of the cylindrically polarized LG modes ${u}_{l,p}^{{\rm{CPLG}}}$ is described in the subsection of Cylindrically polarized Laguerre–Gaussian modes, in Methods.

From Eq. (6), the mode coefficients are

9 $${c}_{l,p}^{r}={\int }_{0}^{R}\,{\int }_{0}^{2\pi }\,({\tilde{E}}_{+}^{{\rm{FR}}}{e}^{{\rm{i}}l\varphi }+{\tilde{E}}_{-}^{{\rm{FR}}}{e}^{{\rm{i}}(\delta -l\varphi )}){({u}_{l,p}^{{\rm{CPLG}}})}^{\ast }{\rm{d}}S,$$

10 $${c}_{l,p}^{\varphi }={\rm{i}}{\int }_{0}^{R}\,{\int }_{0}^{2\pi }\,({\tilde{E}}_{+}^{{\rm{FR}}}{e}^{{\rm{i}}l\varphi }-{\tilde{E}}_{-}^{{\rm{FR}}}{e}^{{\rm{i}}(\delta -l\varphi )}){({u}_{l,p}^{{\rm{CPLG}}})}^{\ast }{\rm{d}}S,$$

satisfying a normalized condition ${\sum }_{l,p}(|{c}_{l,p}^{r}{|}^{2}+|{c}_{l,p}^{\varphi }{|}^{2})=1$. Thus, we can discuss proportions of modes in the complex amplitude vector distribution through the mode coefficients ${c}_{l,p}^{r}$ and ${c}_{l,p}^{\varphi }$. Strictly speaking, the decomposition result is equivalent to the mode distribution at *z* = *z*_0_ when an iris whose diameter is 2*R* is placed at *z* = *z*_0_. We note that the mode coefficients depend on the beam waist radius *w*_0_ and the distance from the beam waist *z*_0_ − *z*_w_, which are described in the subsection of Cylindrically polarized Laguerre–Gaussian modes, in Methods (*z*_w_ is a position of the beam waist on the *z* axis). We set *z*_0_ − *z*_w_ = 0 because the object beam was almost collimated. We describe how we decided the beam waist radius in the next subsection.

### Results and discussions

In this subsection, we describe decomposition results of the complex amplitude distribution into cylindrically polarized LG modes.

**Figure 7:**
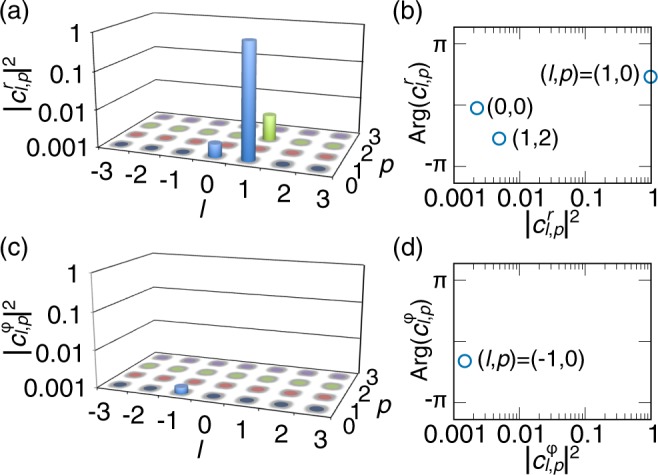
Mode decomposition results for *p* = 0 cylindrically polarized LG pulses for the complex amplitude distributions shown in Fig. 4(a). (**a,c**) Normalized, (*l*, *p*)-resolved intensity $|{c}_{l,p}^{r}{|}^{2}$ and $|{c}_{l,p}^{\varphi }|$ of (**a**) radially polarized and (**c**) azimuthally polarized states, respectively. (**b,d**) (*l*, *p*)-resolved phase ${\rm{Arg}}({c}_{l,p}^{r})$ and ${\rm{Arg}}({c}_{l,p}^{\varphi })$ of (**b**) radially polarized and (**d**) azimuthally polarized states, respectively (the (*l*, *p*)-resolved phase is shown for only the main (*l*, *p*) components, as a function of $|{c}_{l,p}^{r,\varphi }{|}^{2}$).

Figure 7 shows the cylindrically polarized LG mode decomposition results of the complex amplitude distribution of *p* = 0 radially polarized LG pulses [Fig. 4(a)]. Since we intended to generate (*l*, *p*) = (1, 0) radially polarized LG state, we searched the best *w*_0_ maximizing the intensity of the (*l*, *p*) = (1, 0) mode coefficients $|{c}_{\mathrm{1,0}}^{r,\varphi }{|}^{2}$. Its value was *w*_0_ = 202 μm. Since ${c}_{l,p}^{r,\varphi }$ are complex numbers, we show their (*l*, *p*)-resolved intensity of radially polarized states [$|{c}_{l,p}^{r}{|}^{2}$; Fig. 7(a)] and azimuthally polarized states [$|{c}_{l,p}^{\varphi }{|}^{2}$; Fig. 7(c)], and their (*l*, *p*)-resolved phase of radially polarized states [${\rm{Arg}}({c}_{l,p}^{r})$; Fig. 7(b)] and azimuthally polarized states [${\rm{Arg}}({c}_{l,p}^{\varphi })$; Fig. 7(d)] (the (*l*, *p*)-resolved phase is shown for only the main (*l*, *p*) components, as a function of $|{c}_{l,p}^{r,\varphi }{|}^{2}$). The (*l*, *p*)-resolved intensity apparently shows that the object beam was mainly a (*l*, *p*) = (1, 0) radially polarized state. The purity was $|{c}_{\mathrm{1,0}}^{r,\varphi }{|}^{2}=0.981$. The other mode intensities were not zero but less than 0.005. We intended to generate an (*l*, *p*) = (1, 0) radially polarized state, and thus we succeeded in the modulation to the target cylindrically polarized beam with high purity. Thereby, we can quantitatively analyze the quality of cylindrically polarized beams.

**Figure 8:**
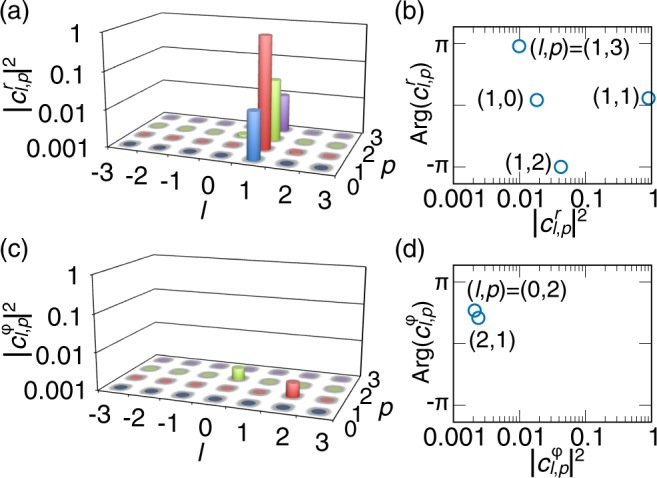
Mode decomposition results for *p* = 1 cylindrically polarized LG pulses for the complex amplitude distributions shown in Fig. 5(a). (**a**,**c**) Normalized, (*l*, *p*)-resolved intensity $|{c}_{l,p}^{r}{|}^{2}$ and $|{c}_{l,p}^{\varphi }|$ of (a) radially polarized and (**c**) azimuthally polarized states, respectively. (**b,d**) (*l*, *p*)-resolved phase ${\rm{Arg}}({c}_{l,p}^{r})$ and ${\rm{Arg}}({c}_{l,p}^{\varphi })$ of (**b**) radially polarized and (**d**) azimuthally polarized states, respectively (the (*l*, *p*)-resolved phase is shown for only the main (*l*, *p*) components, as a function of $|{c}_{l,p}^{r,\varphi }{|}^{2}$).

We decomposed the complex amplitude distribution of *p* = 1 radially polarized LG pulses shown in Fig. 5 into cylindrically polarized LG modes with the same beam waist radius as the (*l*, *p*) = (1, 0) radially polarized beam. We display their (*l*, *p*)-resolved intensity of radially polarized states [$|{c}_{l,p}^{r}{|}^{2}$; Fig. 8(a)] and azimuthally polarized states [$|{c}_{l,p}^{\varphi }{|}^{2}$; Fig. 8(c)], and their (*l*, *p*)-resolved phase of radially polarized states [${\rm{Arg}}({c}_{l,p}^{r})$; Fig. 8(b)] and azimuthally polarized states [${\rm{Arg}}({c}_{l,p}^{\varphi })$; Fig. 8(d)] (the (*l*, *p*)-resolved phase is shown for only the main (*l*, *p*) components, as a function of $|{c}_{l,p}^{r,\varphi }{|}^{2}$.). From the (*l*, *p*)-resolved intensity in Fig. 8(a,c), the (*l*, *p*) = (1, 1) radially polarized LG mode was the dominant one ($|{c}_{\mathrm{1,1}}^{r,\varphi }{|}^{2}=0.904$). The generated beam well agreed with the target beam state ((*l*, *p*) = (1, 1) radially polarized LG mode) whereas there were somewhat ignorable other unwanted modes. The unwanted modes mainly appeared on $l=1$ radially polarized modes [Fig. 8(a)], which indicated that the polarization distribution of the object beam was an $l=1$ radially polarized state but the complex amplitude distribution on the radial axis was deviated from that of a (*l*, *p*) = (1, 1) radially polarized LG mode. The contamination of other modes was ascribed to that a spatial filter (PH in Fig. 8 of ref.^42^) in front of a CCD camera to improve the beam rotational symmetry had a slightly small hole and excited $l=1$, *p* ≠ 1 radially polarized modes. The cylindrically polarized LG mode decomposition thus offers us information in order to discuss experimental results in detail.

In general, the VBSs generated by spatial light modulators, spiral phase plates, or space variant wave plates are Hypergeometric Gaussian modes^62,63^. In contrast, our generation system modulates the intensity pattern on the radial axis by using SLMs so that we can generate LG modes but not Hypergeometric Gaussian modes^42^. Thus, we choose cylindrically polarized LG modes as the base functions.

## Conclusion

We demonstrated the reconstruction of complex amplitude vector distributions of VBSs through the VFR technique and the cylindrically polarized LG mode decomposition of them. The reduction of the number of interferometric image in comparison with the earlier studies^44–46^ enabled us the reconstruction by using an unstable interferometer even with arm length over 6 m. We evaluated accuracy of VFR technique through comparing the ESPs calculated from the polarization distributions to the ones acquired by the IP method. The difference between them was less than 1 degree on the extended Poincaré sphere. Since the VFR method is the integration of interferometry and the IP method, the VFR gives not only spatial polarization profiles of vector beam states but also their spatial phase profiles with high accuracy. We showed that the cylindrically polarized LG modal decomposition of VBSs is meaningful for the detail discussion of experimental results, such as analyses of mode purities and mode contaminations.

The radial index *p*, which gives a node number of a cylindrically polarized LG mode on the radial axis, had been nearly forgotten or regarded as a trivial feature^64^. However, it is theoretically and experimentally proved that the radial index *p* as well as the azimuth index *l* of LG modes is attributed to a quantum number of photon^64–67^. Thus, we note that the full quantitative characterization of VBSs is significant for not only classical optics but also quantum optics.

This VFR and modal decomposition techniques are utilized in mode distribution analysis of lasers emitting VBSs^51,68–72^ and VBS converters^73–79^. Moreover, in the context that some researches^80–82^ claim that multimode waveguides and fibers should be treated with exact modes, that is VBSs, instead of LP modes. Our VFR methods can be also suitable for characterization of spatial mode properties of multimode waveguides and fibers.

## Methods

### Rotating-retarder type imaging polarimetry

We review the method of rotating-retarder type imaging polarimetry^39–41^. The Stokes vector **S** = (*S*_0_, *S*_1_, *S*_2_, *S*_3_)^T^, where *S*_*i*_ (*i* = 0 − 3) are the Stokes parameters, is defined by

11 $${\bf{S}}(x,y,z)=\langle \psi |{\boldsymbol{\sigma }}|\psi \rangle .$$

Here, (*x*, *y*, *z*) represents the Cartesian coordinate, $|\psi \rangle =({\tilde{E}}_{+},{\tilde{E}}_{-}{)}^{{\rm{T}}}$, and **σ** = (*σ*_0_, *σ*_1_, *σ*_2_, *σ*_3_)^T^ is the Pauli spin matrices^83,84^.

We built a rotating-retarder type imaging polarimeter consisting of a quarter-wave plate, a polarizer and a CCD camera (Fig. 1(c)). The polarization axis of the polarizer is parallel to the *y* axis. The complex amplitude distribution at the imaging plane is written by

12 $${\tilde{E}}_{\theta }(x,y,{z}_{0})=\frac{1}{\sqrt{2}}[({\rm{i}}-{e}^{{\rm{i}}2\theta }){\tilde{E}}_{+}-({\rm{i}}-{e}^{-{\rm{i}}2\theta }){\tilde{E}}_{-}]\mathrm{.}$$

We here note that ${\tilde{E}}_{\theta =\pi \mathrm{/4}}={\rm{i}}\sqrt{2}{\tilde{E}}_{-}$ (the LCP component) and ${\tilde{E}}_{\theta =3\pi \mathrm{/4}}=-\,{\rm{i}}\sqrt{2}{\tilde{E}}_{+}$ (the RCP component). Therefore, the intensity distribution recorded by the CCD camera *I*(*x*, *y*, *z*_0_) is described as

13 $${I}_{\theta }(x,y,{z}_{0})=\frac{1}{2}({S}_{0}-{S}_{1}\,{\cos }^{2}\,2\theta -{S}_{2}\frac{1}{2}\,\sin \,4\theta -{S}_{3}\,\sin \,2\theta )\mathrm{.}$$

Since the Stokes parameters have different cycles against the rotational angle *θ* each other, we can acquire each Stokes parameter distribution by measuring images of over four different *θ*. In the experiments, we captured four intensity distributions of *θ* = *π*/8, *π*/4, 3*π*/4 and 7*π*/8. The Stokes vector is described as follows:

14 $${\bf{S}}(x,y,{z}_{0})=(\begin{array}{cccc}0 & 1 & 1 & 0\\ -2 & 2 & 2 & -2\\ 2 & -\sqrt{2} & \sqrt{2} & -2\\ 0 & 1 & -1 & 0\end{array})(\begin{array}{c}{I}_{\theta =\pi \mathrm{/8}}\\ {I}_{\theta =\pi \mathrm{/4}}\\ {I}_{\theta =3\pi \mathrm{/4}}\\ {I}_{\theta =7\pi \mathrm{/8}}\end{array})\mathrm{.}$$

We, thereby, obtain the Stokes parameter distributions on the beam cross section at the measurement position. Here we choose $\theta =\pi /8,\pi /4,3\pi /4$ and 7*π*/8 because *S*_1−3_ are described by the linear combinations of the differences of two intensity distributions at $\theta =\pi /8,\pi /4,3\pi /4$ and 7*π*/8.

### Field reconstruction of uniformly polarized optical vortex states

We briefly review the method of field reconstruction of uniformly optical vortex states^47^. We assume that a *y*-polarized object beam propagating on the *z* axis interferes with a *y*-polarized reference beam propagating on the **k**_0_(*ω*) = *k*(*ω*)(sin*θ*_in_**x** + cos*θ*_in_**z**) direction, where **k**_0_ is a wave vector of the reference beam and **x** and **z** represent the unit vectors of *x* and *z* axes in the Cartesian coordinate. When the bandwidths of the object beam and the reference beam are narrow enough, the electric fields of the object beam **E**_obj_ and the reference beam **E**_ref_ at the measurement plane *z* = *z*_0_ can be approximately written by

15 $${{\bf{E}}}_{{\rm{obj}}}(x,y,{z}_{0},t)\simeq {\tilde{E}}_{{\rm{obj}}}(x,y,{z}_{0})\,\exp \,[{\rm{i}}(k({\omega }_{0}){z}_{0}-{\omega }_{0}t)]{\bf{y}},$$

16 $${{\bf{E}}}_{{\rm{ref}}}(x,y,{z}_{0},y)\simeq {\tilde{E}}_{{\rm{ref}}}(x,y,{z}_{0})\,\exp \,[{\rm{i}}(k({\omega }_{0})\,\cos \,{\theta }_{{\rm{in}}}{z}_{0}-k({\omega }_{0})\,\sin \,{\theta }_{{\rm{in}}}y-{\omega }_{0}t+\alpha )]{\bf{y}},$$

where ${\tilde{E}}_{{\rm{obj}}}$ and ${\tilde{E}}_{{\rm{ref}}}$ are, respectively, the complex amplitude of the object and the reference beams, *ω*_0_ is the center wavelength of the object and the reference beams, and *α* is a constant phase between the object and the reference beams. The two dimentional Fourier transform (2D-FT) of the interference pattern |**E**_obj_ + **E**_ref_|^2^ consists of four terms:

17 $$ {\mathcal F} (|{{\bf{E}}}_{{\rm{obj}}}+{{\bf{E}}}_{{\rm{ref}}}{|}^{2})= {\mathcal F} (|{\tilde{E}}_{{\rm{obj}}}{|}^{2})+ {\mathcal F} (|{\tilde{E}}_{{\rm{ref}}}{|}^{2})+{I}_{{\rm{AC}}+}+{I}_{{\rm{AC}}-},$$

where ${\mathcal F} (\cdot )$ denotes the 2D-FT in the (*x*, *y*) plane. The first and the second terms are, respectively, the 2D-FTs of the intensity distributions of the object and the reference beams, whose intensity peaks appears on the origin of the wavenumber space (*k*_*x*_, *k*_*y*_) = (0, 0). The third and the fourth ones are described as

18 $${I}_{{\rm{AC}}+}={I}_{{\rm{AC}}-}^{\ast }= {\mathcal F} \{{\tilde{E}}_{{\rm{obj}}}\,\exp \,({\rm{i}}k\,\sin \,{\theta }_{{\rm{in}}}y){\tilde{E}}_{{\rm{ref}}}^{\ast }\}\exp \{{\rm{i}}k\mathrm{(1}-\,\cos \,{\theta }_{{\rm{in}}}){z}_{0}-\alpha \}\mathrm{.}$$

The peaks of the intensity of |*I*_AC±_|^2^ in the wavenumber space are at (*k*_*x*_, *k*_*y*_) = (0, ±*k*sin*θ*_in_), respectively. Filtering around the (*k*_*x*_, *k*_*y*_) = (0, +*k*sin*θ*_in_), we can extract the third term having information of the complex amplitude component of the object beam. The size of the reference beam we used in experiments was estimated to be 3 mm, which is large (over ~5 times larger than the size of the object beam) enough to consider that ${\tilde{E}}_{{\rm{ref}}}$ was constant. Hence, a 2D-FT of the complex amplitude of the object beam ${\tilde{E}}_{{\rm{obj}}}^{{\rm{FT}}}({k}_{x},{k}_{y},{z}_{0})= {\mathcal F} ({\tilde{E}}_{{\rm{obj}}})$ is described as

19 $${\tilde{E}}_{{\rm{obj}}}^{{\rm{FT}}}({k}_{x},{k}_{y}-k\,\sin \,{\theta }_{{\rm{in}}},{z}_{0})={I}_{{\rm{AC}}+}\,\exp \,(-{\rm{i}}\beta )/|{\tilde{E}}_{{\rm{ref}}}|,$$

where *β* = (*k* − *k*_0*z*_)*z*_0_ − *α* is a constant phase. Thereby, the complex amplitude of the object beam ${\tilde{E}}_{{\rm{obj}}}^{{\rm{FR}}}(x,y,{z}_{0})$ is reconstructed by using the two dimentional inverse Fourier transform ${ {\mathcal F} }^{-1}(\cdot )$:

20 $${\tilde{E}}_{{\rm{obj}}}^{{\rm{FR}}}(x,y,{z}_{0})\equiv { {\mathcal F} }^{-1}\{{\tilde{E}}_{{\rm{obj}}}^{{\rm{FT}}}({k}_{x},{k}_{y},{z}_{0})\}\mathrm{.}$$

We note that *β* is indefinite constant because *α* is easy to fluctuate depending on the optical path difference owing to unstableness of an interferometer. Here, the relative phase *δ* is defined by *δ* = *β*_−_ − *β*_+_, where *β*_+_ and *β*_−_ are the constant phases *β* for reconstructed RCP and LCP complex amplitude components (${\tilde{E}}_{+}^{{\rm{FR}}}$ and ${\tilde{E}}_{-}^{{\rm{FR}}}$), respectively.

### Extended Stokes parameters

We here review the ESPs. See the supplemental material of refs^40,41^ for the detail. The ESPs obtained by the IP method are defined by the following equation:

21 $$(\begin{array}{c}{S}_{\mathrm{0,}l}^{{\rm{E}}}\\ {S}_{\mathrm{1,}l}^{{\rm{E}}}\\ {S}_{\mathrm{2,}l}^{{\rm{E}}}\\ {S}_{\mathrm{3,}l}^{{\rm{E}}}\end{array})={\iint }_{A}\,(\begin{array}{c}{S}_{0}(\tilde{x},\tilde{y})\\ {S}_{1}(\tilde{x},\tilde{y})\,\cos \,\mathrm{(2}l\varphi )+{S}_{2}(\tilde{x},\tilde{y})\,\sin \,\mathrm{(2}l\varphi )\\ -{S}_{1}(\tilde{x},\tilde{y})\,\sin \,\mathrm{(2}l\varphi )+{S}_{2}(\tilde{x},\tilde{y})\,\cos \,\mathrm{(2}l\varphi )\\ {S}_{3}(\tilde{x},\tilde{y})\end{array}){\rm{d}}\tilde{x}{\rm{d}}\tilde{y},$$

where *A* is an area of interest, and *l* is the azimuthal index of the ESPs. In the present paper, we regarded the area as a circle, whose center was (*x*, *y*) = (*c*_*x*_, *c*_*y*_). Modified coordinates $(\tilde{x},\tilde{y})$ are (*x* − *c*_*x*_, *y* − *c*_*y*_). The azimuthal angle *ϕ* is described by $\arctan (\tilde{y}/\tilde{x})$. Here, the component of temporally-perfect-polarized state in ${S}_{\mathrm{0,}l}^{{\rm{E}}}$ are described by

22 $${S}_{\mathrm{0,}l}^{E,(P)}={\iint }_{A}\,{\{{({S}_{1})}^{2}+{({S}_{2})}^{2}+{({S}_{3})}^{2}\}}^{\mathrm{1/2}}{\rm{d}}\tilde{x}{\rm{d}}\tilde{y}.$$

Unlike higher-order^85–87^ and hybrid-order^88^ Stokes parameters, the ESPs are capable of the definition of the degree of polarization (DOP). The DOP for the ESPs is defined by

23 $${{\mathscr{P}}}_{l}^{{\rm{space}}}={\{{({S}_{\mathrm{1,}l}^{{\rm{E}}})}^{2}+{({S}_{\mathrm{2,}l}^{{\rm{E}}})}^{2}+{({S}_{\mathrm{3,}l}^{{\rm{E}}})}^{2}\}}^{\mathrm{1/2}}/{S}_{\mathrm{0,}l}^{E,(P)},$$

which gives a measure of symmetry in the *l*th cylindrically polarized state. The normalized ESPs (or temporally- and spatially-perfect-polarized Stokes vector) are described by

24 $${\tilde{{\bf{S}}}}_{l}^{{\rm{E}}}=(\begin{array}{c}{\tilde{S}}_{\mathrm{1,}l}^{{\rm{E}}}\\ {\tilde{S}}_{\mathrm{2,}l}^{{\rm{E}}}\\ {\tilde{S}}_{\mathrm{3,}l}^{{\rm{E}}}\end{array})\equiv \frac{1}{{S}_{\mathrm{0,}l}^{E,(P)}{{\mathscr{P}}}_{l}^{{\rm{space}}}}(\begin{array}{c}{S}_{\mathrm{1,}l}^{{\rm{E}}}\\ {S}_{\mathrm{2,}l}^{{\rm{E}}}\\ {S}_{\mathrm{3,}l}^{{\rm{E}}}\end{array})\mathrm{.}$$

Since $|{\tilde{{\bf{S}}}}_{l}^{{\rm{E}}}|\mathrm{=1}$, the normalized extended Stokes vector is always on the extended Poincaré sphere.

Here, we searched the center of the area *A* maximizing the $l=1$ DOP ${{\mathscr{P}}}_{l}^{{\rm{space}}}$. The radius of the circle (the cutoff radius) *R* was set to be Max[*I*(*r*)]/100 = *I*(*R*), where

25 $$I(R)={\int }_{0}^{R}{\int }_{0}^{2\pi }{\{{({S}_{1})}^{2}+{({S}_{2})}^{2}+{({S}_{3})}^{2}\}}^{\mathrm{1/2}}{\rm{d}}\varphi {\rm{d}}r\mathrm{.}$$

The values of *R* for the *p* = 0 and *p* = 1 radially polarized LG pulses were 387 and 516 μm, repsectively.

Similarly, we define the ESPs derived from the VFR method, the DOP for the ESPs, and the normalized ESPs by

26 $$(\begin{array}{c}{S}_{\mathrm{0,}l}^{E,\mathrm{VFR}}\\ {S}_{\mathrm{1,}l}^{E,\mathrm{VFR}}\\ {S}_{\mathrm{2,}l}^{E,\mathrm{VFR}}\\ {S}_{\mathrm{3,}l}^{E,\mathrm{VFR}}\end{array})={\iint }_{A}\,(\begin{array}{c}{|{\tilde{E}}_{+}^{{\rm{FR}}}|}^{2}+{|{\tilde{E}}_{-}^{{\rm{FR}}}|}^{2}\\ 2\Re \{{({\tilde{E}}_{+}^{{\rm{FR}}})}^{\ast }{\tilde{E}}_{-}^{{\rm{FR}}}{e}^{{\rm{i}}(\delta -2l\varphi )}\}\\ 2\Im \{{({\tilde{E}}_{+}^{{\rm{FR}}})}^{\ast }{\tilde{E}}_{-}^{{\rm{FR}}}{e}^{{\rm{i}}(\delta +2l\varphi )}\}\\ {|{\tilde{E}}_{+}^{{\rm{FR}}}|}^{2}-{|{\tilde{E}}_{-}^{{\rm{FR}}}|}^{2}\end{array}){\rm{d}}\tilde{x}{\rm{d}}\tilde{y},$$

27 $${{\mathscr{P}}}_{l}^{\mathrm{space},\mathrm{VFR}}={\{{({S}_{\mathrm{1,}l}^{E,\mathrm{VFR}})}^{2}+{({S}_{\mathrm{2,}l}^{E,\mathrm{VFR}})}^{2}+{({S}_{\mathrm{3,}l}^{E,\mathrm{VFR}})}^{2}\}}^{\mathrm{1/2}},$$

and

28 $${\tilde{{\bf{S}}}}_{l}^{E,\mathrm{VFR}}=(\begin{array}{c}{\tilde{S}}_{\mathrm{1,}l}^{E,\mathrm{VFR}}\\ {\tilde{S}}_{\mathrm{2,}l}^{E,\mathrm{VFR}}\\ {\tilde{S}}_{\mathrm{3,}l}^{E,\mathrm{VFR}}\end{array})\equiv \frac{1}{{S}_{\mathrm{0,}l}^{E,(P),\mathrm{VFR}}{{\mathscr{P}}}_{l}^{{\rm{space}}}}(\begin{array}{c}{S}_{\mathrm{1,}l}^{E,\mathrm{VFR}}\\ {S}_{\mathrm{2,}l}^{E,\mathrm{VFR}}\\ {S}_{\mathrm{3,}l}^{E,\mathrm{VFR}}\end{array}),$$

respectively. We note that ${S}_{\mathrm{0,}l}^{E,(P),\mathrm{VFR}}\equiv {S}_{\mathrm{0,}l}^{E,\mathrm{VFR}}$ because a field reconstruction technique based on interferometry can retrieve only the temporally-perfect-polarized state.

### Evaluation of relative phase *δ*

We here propose a method to evaluate the relative phase *δ*. First, we observe four interference patterns when $\theta =\pi /8,\pi /4,3\pi /4$, and 7*π*/8 by using the polarization analyzer system shown in Fig. 1(c), following which we reconstruct their complex amplitude distributions ${\tilde{E}}_{\theta =\pi \mathrm{/8}}^{{\rm{FR}}}$, ${\tilde{E}}_{\theta =\pi \mathrm{/4}}^{{\rm{FR}}}\equiv {\tilde{E}}_{+}^{{\rm{FR}}}$, ${\tilde{E}}_{\theta \mathrm{=3}\pi \mathrm{/4}}^{{\rm{FR}}}\equiv {\tilde{E}}_{-}^{{\rm{FR}}}$, and ${\tilde{E}}_{\theta \mathrm{=7}\pi \mathrm{/8}}^{{\rm{FR}}}$, respectively. The Stokes parameters **S**^IP,FR^(*x*, *y*, *z*_0_) are calculated from the intensity distribution of them as the IP method (Eq. (14)):

29 $${{\bf{S}}}^{\mathrm{IP},\mathrm{FR}}(x,y,{z}_{0})=(\begin{array}{cccc}0 & 1 & 1 & 0\\ -2 & 2 & 2 & -2\\ 2 & -\sqrt{2} & \sqrt{2} & -2\\ 0 & 1 & -1 & 0\end{array})(\begin{array}{c}{|{E}_{\theta =\pi \mathrm{/8}}^{{\rm{FR}}}|}^{2}\\ {|{E}_{\theta =\pi \mathrm{/4}}^{{\rm{FR}}}|}^{2}\\ {|{E}_{\theta \mathrm{=3}\pi \mathrm{/4}}^{{\rm{FR}}}|}^{2}\\ {|{E}_{\theta \mathrm{=7}\pi \mathrm{/8}}^{{\rm{FR}}}|}^{2}\end{array})\mathrm{.}$$

The intensity distribution of *x*-polarized component is obtained as

30 $$\begin{array}{rcl}{|{E}_{x}^{{\rm{IP}}}(r,\varphi ,{z}_{0})|}^{2} & = & \frac{1}{2}({S}_{0}^{\mathrm{IP},\mathrm{FR}}+{S}_{1}^{\mathrm{IP},\mathrm{FR}})\\  & = & \begin{array}{c}-\frac{1}{2}(-{|{\tilde{E}}_{\theta =\pi \mathrm{/4}}^{{\rm{FR}}}|}^{2}+{|{\tilde{E}}_{\theta =3\pi \mathrm{/4}}^{{\rm{FR}}}|}^{2})\\ +{|{\tilde{E}}_{\theta =\pi \mathrm{/8}}^{{\rm{FR}}}|}^{2}+{|{\tilde{E}}_{\theta =7\pi \mathrm{/8}}^{{\rm{FR}}}|}^{2}\mathrm{.}\end{array}\end{array}$$

By using Eq. (4), we search *δ* which minimizes *G*_*δ*_. The center position (*r* = 0) was selected to be the position maximizing the DOP-SD^38,40,41,43^ ${{\mathscr{P}}}_{l=1}^{(\mathrm{space})}$. The definition of DOP-SD is described in the subsection of Extended Stokes parameters. We note that if we measure a *y*-polarized component instead of ${|{E}_{\theta =\pi \mathrm{/8}}^{{\rm{FR}}}|}^{2}$ and ${|{E}_{\theta \mathrm{=7}\pi \mathrm{/8}}^{{\rm{FR}}}|}^{2}$, we can reduce the total number of acquisition images because ${|{E}_{y}^{{\rm{IP}}}(r,\varphi ,{z}_{0})|}^{2}={|{E}_{\theta \mathrm{=0}}^{{\rm{FR}}}|}^{2}$. In this case, *δ* and ${|{E}_{x}^{{\rm{IP}}}(r,\varphi ,{z}_{0})|}^{2}$ in Eqs (3), (4), (9) and (10) should be replaced by *δ* + *π* and ${|{E}_{y}^{{\rm{IP}}}(r,\varphi ,{z}_{0})|}^{2}$, respectively.

### Cylindrically polarized Laguerre–Gaussian modes

As Eq. (6), cylindrically polarized LG modes are described by *l*-topological-charge LG modes with *p*th radial index without the phase ramp^50^:

31 $$\begin{array}{c}{u}_{l,p}^{{\rm{CPLG}}}(r,\varphi ,z)=\sqrt{\frac{2p!}{\pi (p+|l|)!}}{(\frac{\sqrt{2}r}{w})}^{|l|}{L}_{p}^{|l|}(\frac{2{r}^{2}}{{w}^{2}})\frac{{w}_{0}}{w}\\ \,\,\,\,\,\,\times \exp [-\frac{{r}^{2}}{{w}^{2}}+{\rm{i}}(k\frac{{r}^{2}}{2R(z)}-{\psi }_{l,p}^{{\rm{Gouy}}}({z}_{0}))],\end{array}$$

where ${L}_{p}^{|l|}(\xi )$ represents the generalized Laguerre polynomials. *w*, *R*(*z*), and *ψ*^Gouy^(*z*) are, respectively, a beam size at *z* and a radius of curvature at *z*, and the Gouy phase, which are described by

32 $$w={w}_{0}{(1+\frac{{(z-{z}_{{\rm{w}}})}^{2}}{{z}_{{\rm{R}}}^{2}})}^{\mathrm{1/2}},$$

33 $$R(z)=\frac{{(z-{z}_{{\rm{w}}})}^{2}+{z}_{{\rm{R}}}^{2}}{z-{z}_{{\rm{w}}}},$$

34 $${\psi }_{l,p}^{{\rm{Gouy}}}(z)=(|l|+2p+\mathrm{1)}\arctan (\frac{z-{z}_{{\rm{w}}}}{{z}_{{\rm{R}}}}),$$

where *w*_0_ = (2*z*_R_/*k*)^1/2^ is a beam waist radius, *z*_w_ is a position of the beam waist on the *z* axis, and *z*_R_ is the Rayleigh length. The cylindrically polarized LG modes described in Eq. (31) are normalized.

35 $${\iint }_{r,\varphi }\,{|{u}_{l,p}^{{\rm{CPLG}}}(r,\varphi ,z)|}^{2}{\rm{d}}S=1.$$
